# Clinical Effectiveness and Cost-effectiveness of Videoconference-Based Integrated Cognitive Behavioral Therapy for Chronic Pain: Randomized Controlled Trial

**DOI:** 10.2196/30690

**Published:** 2021-11-22

**Authors:** Kayoko Taguchi, Noriko Numata, Rieko Takanashi, Ryo Takemura, Tokiko Yoshida, Kana Kutsuzawa, Kensuke Yoshimura, Natsuko Nozaki-Taguchi, Seiji Ohtori, Eiji Shimizu

**Affiliations:** 1 Research Center for Child Mental Development Chiba University Chiba Japan; 2 Department of Psychology Teikyo University Hachioji Japan; 3 Keio University Hospital Tokyo Japan; 4 Graduate School of Medicine Chiba University Chiba Japan; 5 Chiba University Hospital Chiba Japan

**Keywords:** cognitive behavioral therapy, chronic pain, medical economic evaluation, EQ-5D-5L, telemedicine

## Abstract

**Background:**

Cognitive behavioral therapy is known to improve the management of chronic pain. However, the components of this therapy are still being investigated and debated.

**Objective:**

This study aimed to examine the effectiveness of an integrated cognitive behavioral therapy program with new components (attention-shift, memory work, video feedback, and image training) delivered via videoconferencing.

**Methods:**

This study was unblinded and participants were recruited and assessed face-to-face in the outpatient department. We conducted a randomized controlled trial for chronic pain to compare 16 weekly videoconference-based cognitive behavioral therapy (vCBT) sessions provided by a therapist with treatment as usual (TAU). Thirty patients (age range, 22-75 years) with chronic pain were randomly assigned to either vCBT (n=15) or TAU (n=15). Patients were evaluated at week 1 (baseline), week 8 (midintervention), and week 16 (postintervention). The primary outcome was the change in pain intensity, which was recorded using the numerical rating scale at 16 weeks from the baseline. Secondary outcomes were pain severity and pain interference, which were assessed using the Brief Pain Inventory. Additionally, we evaluated disability, pain catastrophizing cognition, depression, anxiety, quality of life, and cost utility.

**Results:**

In the eligibility assessment, 30 patients were eventually randomized and enrolled; finally, 15 patients in the vCBT and 14 patients in the TAU group were analyzed. Although no significant difference was found between the 2 groups in terms of changes in pain intensity by the numerical rating scale scores at week 16 from baseline (*P*=.36), there was a significant improvement in the comprehensive evaluation of pain by total score of Brief Pain Inventory (–1.43, 95% CI –2.49 to –0.37, *df*=24; *P*=.01). Further, significant improvement was seen in pain interference by using the Brief Pain Inventory (–9.42, 95% CI –14.47 to –4.36, *df*=25; *P*=.001) and in disability by using the Pain Disability Assessment Scale (–1.95, 95% CI –3.33 to –0.56, *df*=24; *P*=.008) compared with TAU. As for the Medical Economic Evaluation, the incremental cost-effectiveness ratio for 1 year was estimated at 2.9 million yen (about US $25,000) per quality-adjusted life year gained.

**Conclusions:**

The findings of our study suggest that integrated cognitive behavioral therapy delivered by videoconferencing in regular medical care may reduce pain interference but not pain intensity. Further, this treatment method may be cost-effective, although this needs to be further verified using a larger sample size.

**Trial Registration:**

University Hospital Medical Information Network UMIN000031124; https://tinyurl.com/2pr3xszb

## Introduction

Global reports indicate that chronic pain affects an estimated 20%-50% of people [1-4]. The COVID-19 pandemic has made it more difficult for people with chronic pain to safely visit hospitals. Chronic pain results in significant economic losses, with studies suggesting that pain management imposes a substantial burden on health care resources worldwide [5,6]. In Japan, it has been reported that economic loss from the inability to work due to pain is approximately 1.8 trillion yen per year [7]. It is, therefore, necessary to establish an urgent treatment system to benefit such patients and, in turn, the nation. Although multidisciplinary pain management is recommended as effective treatment for chronic pain, in clinical practice, psychosocial approaches to chronic pain are generally considered a last resort, thus delaying psychosocial intervention. Therefore, one can say that psychosocial approaches to chronic pain are underdeveloped or insufficient [8].

Cognitive behavioral therapy (CBT) is a structural intervention that encourages the transformation of a patient’s cognition and behavior and directly addresses major psychological problems associated with chronic pain, such as repetitive ideation, concerns, emotions, and behaviors. CBT is recognized and recommended as an effective treatment for managing chronic pain [9]. The latest review on the effect of CBT verified by randomized controlled trials (RCTs) has added 41 new studies to the existing 34 studies, thus creating a large pool of 75 verified RCTs. Comparisons of CBT with active controls showed a slight benefit in terms of pain intensity, disability, and distress immediately after treatment [10]. It was also observed that there were small merits in each of the 3 outcomes as compared to no treatment. At follow-up, pain, disability, distress, and other variables were maintained in comparison with no treatment, but there is a lack of rigorous studies that involve active controls [10]. In summary, there is evidence of efficacy, although the effect size of CBT for chronic pain is small and insufficient.

The use of remote treatment across the internet has been increasing and its effectiveness has been demonstrated for various intractable diseases [11-14]. Internet-based CBT conducted for chronic pain has been on the rise [15-18]. While such remote treatment is known by several names such as internet-delivered CBT, web-based CBT, and telemedicine, these are all strictly different interventions. Web-based CBT and internet-delivered CBT were used synonymously in a study in which patients performed CBT on their own as self-help training or received regular therapist feedback that was not face-to-face [19,20]. Moreover, many studies involved treatment programs delivered via the internet or through smartphone apps, with the aim of self-management by patients (no intervention by the therapist) [21-23].

Only a few studies have examined the effectiveness of videoconference-based CBT (vCBT), which is face-to-face CBT using a videoconference system and not a general internet-delivered CBT for chronic pain management [24-26]. The aim of this study was to verify the cost and clinical effectiveness of a new integrated CBT program for chronic pain, delivered through videoconferencing (vCBT), and to compare with treatment as usual (TAU) for pain intensity, pain interference, disability, pain-related catastrophizing cognition, depression, anxiety, quality of life, and cost utility.

## Methods

### Study Design, Setting, and Participants

This study was designed as a prospective randomized unblinded pilot trial comparing vCBT as the intervention group to TAU as the control group at the academic outpatient clinic of the Cognitive Behavioral Therapy Center at Chiba University Hospital. Patients with intractable chronic pain, aged 18-75 years, were randomized and enrolled in one of the two groups. The intervention period was 16 weeks for both groups. The vCBT group received 1 weekly session of an integrated CBT program in addition to regular medical care. The TAU group continued outpatient consultation more than once for 8 weeks. For primary and secondary outcomes, patients were evaluated at week 1 (baseline), week 8 (midintervention), and week 16 (postintervention).

Although it is difficult to define intractable chronic pain, it is considered “drug-resistant” because almost all patients with acute pain receive pharmacotherapy. A review of evidence-based clinical trial designs for chronic pain pharmacotherapy states that “regulatory agencies such as the United States Food and Drug Administration and European Medicines Agency require studies of 12-weeks duration for chronic pain such as neuropathic pain to demonstrate the durability of response” [27]. Another review states that “some longer duration trials have shown efficacy of the investigational medication early in the course of treatment, only to lose statistical significance as the placebo group catches up” [28]. Consequently, the appropriate period of drug resistance cannot be determined, although taking these previous studies into account, the condition of drug resistance can be thought of as one in which individuals with chronic pain do not show moderate to remarkable improvement owing to poor tolerability, despite receiving sufficient pharmacotherapy for at least 12 weeks.

### Ethics and Dissemination

This study was conducted with the approval of the Institutional Review Board of the Chiba University Hospital (approval ID G29049). In addition, the Clinical Research Ethics Review Committee oversaw the proper implementation of the test at least once a year. The trial registration number was University Hospital Medical Information Network UMIN000031124. The patients willing to participate in this study were informed of the study objectives and were asked for their consent to participate. Each patient was informed that participation was voluntary and full anonymity would be provided. Each patient was required to provide written consent for participation.

### Recruitment

We recruited participants through web-based and newspaper advertisements from April 2018 to November 2019. Patient recruitment was announced by the doctors at outpatient clinics in the Department of Orthopedics and Pain Anesthesiology at Chiba University Hospital and in all medical institutions in the Chiba prefecture as well. All recruitment materials referred patients to our study website, which explained the study in detail. All participants who gave their permission to be enrolled in the study were required to continue treatment with their general practitioners as TAU. Patients who were interested in the study could inquire about the details via email. This mail was also used as an application form to ask patients to record their age, sex, condition of chronic pain, contact information, and so forth.

### Eligibility Procedure for Participation and Diagnosis

Written informed consent was obtained face-to-face from all patients after they were fully briefed on the procedure. Following this, a screening eligibility assessment for inclusion and exclusion criteria was performed. Participants were asked to record their pain intensity rated by the Numerical Rating Scale (NRS) daily for a week. Inclusion criteria were as follows: (1) fulfillment of the criteria of somatic symptom disorder, with predominant pain according to the fifth edition of the Diagnostic and Statistical Manual of Mental Disorders (DSM-5), (2) age ranging from 18 to 75 years to avoid the risks from cognitive decline, (3) not showing moderate to remarkable improvement despite receiving sufficient pharmacotherapy for more than 8 weeks or due to poor tolerability, and (4) appropriate mental and physical conditions to maintain therapy. In the case of patients with depression or other anxiety conditions, they met the criteria to take part in the study if their pain was the primary impairment. The exclusion criteria were (1) comorbidity of serious mental disorders such as neurocognitive disorder, psychotic disorder, bipolar disorder, or substance-related disorder based on the criteria in DSM-5; (2) major pain caused by cancer; (3) if their pain did not interfere with their daily life (PDAS: Pain Disability Assessment Scale score of 9 or less); (4) mental retardation, neurocognitive disorders (dementia), and autism spectrum disorder; and (5) litigation or compensation concerning pain symptoms. In this study, patients were required to be able to use a videoconferencing system at home. In case of patients who did not have an internet connection in their houses, we rented tablet computers and mobile Wi-Fi devices for them.

### Randomization and Case Registration

The eligible patients were randomly assigned to either the vCBT group or the TAU group using the minimization method used in clinical trials to ensure a balance in pain intensity score and gender. Per the allocation adjustment factor, the pain intensity score on NRS was allocated at 6.3 for each group [29]. The randomization and assignment were done by the Clinical Research Data Center, and the results were informed to the researchers by fax.

### Intervention

The intervention period was from May 2018 to April 2020. In both groups, patients underwent 16 weeks of intervention and answered questions regarding primary and secondary outcomes at 1 week (baseline), 8 weeks (middle), and 16 weeks (post). All patients continued the regular medical care that they would normally receive as treatment. While participating in this study, no patient was permitted to seek any new treatment other than those that their primary care doctor ordered. In addition to regular medical care, those allocated to the vCBT group received weekly 50-minute sessions over 16 weeks of integrated CBT program using real-time internet videoconferencing. If the therapists or patients found it impossible to continue owing to adverse events, a gap of maximum 30 days during the intervention was permitted.

### Videoconference-Based Integrated CBT Program

The CBT program that we adopted is an integrated CBT program that is longer than conventional interventions and consists of several new sessions not used in traditional CBT protocols. Our developed protocol with face-to-face CBT sessions provided by the videoconference system (web-based CBT) has been shown to improve catastrophic cognition, disability, and mood [30]. Conventional CBT programs for chronic pain often comprise 8-12 intervention sessions. In almost all programs, psychoeducation for pain, case formulation for understanding cognitive behavioral models of chronic pain, relaxation exercises such as breathing, and cognitive reconstruction, among others, were included [31]. Each of the 16 sessions lasted 50 minutes. We added 4 new sessions: tactile attention-shift training (session 4), memory work based on peak-end rule (session 10), sharpening behavioral image training (session 11), and video feedback (session 12) to the conventional CBT program (shown in Table 1).

**Table 1:** Integrated cognitive behavioral therapy program for chronic pain.

Session	Program	Description
Session 1	Introduction	Therapists explained the purpose of cognitive behavioral therapy and set short-, medium-, and long-term treatment goals.
Session 2	Psychoeducation	Patients studied ideas such as the mechanism of pain, gate-control theory, and acceptance of pain.
Session 3	Relaxation	Patients practiced progressive muscle relaxation and abdominal breathing techniques.
Session 4	Tactile attention-shift training	Patients practiced flexibly shifting their excessive attention to pain.
Session 5	Case formulation	Patients learned their own cognitive behavioral models and vicious pain-causing cycles.
Session 6	Safety behaviors	For behavioral activation, patients understood avoiding action due to pain and learned the demerits of continuing safety action such as avoidance, makeshift action.
Session 7	Cognitive restructuring 1	Patients’ thinking habits were examined and they learned how to change their irrational thinking.
Session 8	Cognitive restructuring 2	Patients’ thinking habits were examined and they learned how to change their irrational thinking.
Session 9	Activity pacing	Patients spaced out activities to manage pain.
Session 10	Memory work using the peak end rule	By reexamining their pain memory, patients learned that it influences chronic pain.
Session 11	Mental practice	Patients practiced imagining the movement of their body in pain and maintaining hope.
Session 12	Visual feedback	Patients performed mirror therapy as an alternative, recording own actions and observing ideal movement.
Session 13	Behavioral experiments 1	Patients practiced step by step those actions that could not be performed because of pain.
Session 14	Behavioral experiments 2	Patients practiced step by step those actions that could not be performed because of pain.
Session 15	Summary	We reviewed all the sessions and confirmed any remaining issues.
Session 16	Relapse prevention	Patients learned to think about how to respond when the pain recurred.

Tactile attention-shift training (session 4): Patients with chronic pain tend to demonstrate “attention bias.” Excessive attention causes patients’ pain to increase. Patients were trained to flexibly shift their attention. Furthermore, it has been found that pain may be relieved by gentle strokes that promote the secretion of oxytocin. This is referred to as the Science Touch method and suggests relief from chronic pain [32]. Attention-shift training takes place while being conscious of the sense of touch.

Memory work based on the peak-end rule (session 10): With significant chronic pain, patients tend to consider their memory of pain to be worse than the actual intensity of the pain. This is because the memory of an intense pain experience is saved as a traumatic memory [33]. The peak-end rule theory explains that “unpleasant experiences like pain are memorized as the average of the strength of the pain peak and the strength of the end” [33]. Patients with chronic pain tend to retain the memory of the peak of their pain experience, when the pain was too intense, and they may not be able to remember the end or release of their pain. Their peak (painful) memories are thus surpassed by the end (good) memories. Therefore, in their pain memory, the average degree of the pain and unpleasantness is high, and it remains as an extreme memory where objectivity about the pain condition is lost. This causes negative emotions such as anxiety and depressive feelings, which can be a factor for negative thoughts. This is considered catastrophic cognitionit is caused by pain but at the same time, it becomes the factor of chronicity. This session seeks to reduce pain by recalling memories of intense pain in detail and reconstructing them in objective memory.

Sharpening behavioral image training (session 11): We were inspired by sports training and poststroke rehabilitation [34,35] to compose this session. Many patients with chronic pain cannot imagine that they can move the injured body part since they have not moved it in a long time. Therefore, this session helped patients increase their daily movement using “image training” until they could clearly imagine moving their painful body parts themselves.

Video feedback (session 12): We developed this session in line with the principles of mirror therapy, a treatment used for phantom limb pain [36]. In this therapy, when the patient moves their healthy side, it is reflected in the mirror and the patient visually recognizes the movement. As a result, the brain interprets it as the amputated side that is moving and thus, the pain is alleviated [37,38]. When there is no movement in the painful area for a long time, it is considered to be a state in which the sensory and motor contact with the brain is severely disconnected. This is regarded as being similar to experiencing phantom limb pain. Thus, we considered that if pain can be alleviated by observing repeated normal movements in the mirror, observing normal movements in videos should also have similar effects.

### System Safety

In this study, we adopted the ISO 27001-certified Cisco WebEx as the internet conference system. Countermeasures against unauthorized access, information leakage, and others were taken, and safety problems were cleared.

### Measures and Evaluation

#### Pretreatment Measures

After enrollment, we assessed the baseline characteristics of the patient’s sex, age, education, marital status, comorbidity, employment status, age at onset of pain, duration of pain, and treatment history before they entered the intervention period (shown in Table 2). The following outcome measures were set based on the Initiative on Methods, Measurement, and Pain Assessment in Clinical Trials. Furthermore, “Assessment of Chronic Pain” was recommended by the International Pain Society [39].

**Table 2:** Baseline characteristics of the patients.

Characteristics		Videoconference-based cognitive behavioral therapy (n=15)		Treatment as usual (n=14)
Age (years), mean (SD)		50.0 (14.3)		43.9 (12.5)
Females, n (%)		10 (67)		9 (64)
Education history (years), mean (SD)		13.6 (2.6)		13.4 (3.8)
Currently employed, n (%)		4 (26)		5 (35)
Family members living together, mean (SD)		2.0 (2.1)		1.8 (1.0)
**Chronic pain site, n (%)^a^**				
	Lower back	8 (53)	3 (21)	
	Back	6 (38)	3 (21)	
	Neck	4 (27)	1 (7)	
	Arm	3 (21)	4 (27)	
	Leg	7 (47)	7 (50)	
	Other	8 (53)	2 (14)	
Duration of disease (years), mean (SD)		11.0 (12.6)		7.6 (5.8)
Mental status comorbidity, n (%)		6 (40)		8 (57)

^a^For chronic pain site, duplicate answers were possible.

#### Adverse Events

All adverse events were reported irrespective of their relevance to the intervention of this study, and serious adverse events were immediately reported to the institutional review board of the Chiba University Hospital and registered with the hospital risk management system as well. Moreover, an independent data monitoring committee accurately verified the detailed records of the clinical study’s progress, critical efficacy variables, and safety data.

#### Primary Outcome Measures

The primary outcome was the change in the pain intensity from baseline to week 16, as indicated by the NRS score. The NRS is a self-rated questionnaire that measures pain intensity on a scale of 0-10, where 0=nothing and 10=severe. Patients were made to keep a daily pain diary. They recorded (1) maximum pain throughout the day, (2) minimum pain, (3) usual pain, and calculated the weekly average for pain on the day of the session (each NRS score=sum total of 1-week NRS score/7). Numerical values obtained by averaging the values in (1), (2), and (3) were taken as the main evaluation items comprising the composite value of NRS. The measurement has been shown to be reliable and valid [40].

#### Secondary Outcome Measures

All secondary outcomes were measured at 8 weeks and 16 weeks from the baseline.

Pain intensity: The secondary outcome was change in pain intensity (maximum, minimum, usual score) from baseline to week 8 on the NRS.

Comprehensive pain score: Comprehensive pain was assessed with the Japanese translation of the Brief Pain Inventory (BPI) [41]. BPI is composed of 2 factors: pain severity and pain interference. Pain severity means the pain intensity, but we use the original term as used in the BPI. The scale has a high reliability (coefficient alpha greater than .80) and established validity. Pain severity on the BPI comprises 4 items (worst, least, average, and current). They are assessed as 0 (nothing) to 10 (severe), with higher scores representing worse pain. Pain severity was calculated as the average of the 4 scores. Pain interference of BPI is a 7-item measure designed to assess pain interference by sleep, mood, social relations, and enjoyment of life. On an 11-point scale (0=does not interfere, 10=completely interferes), patients indicated how much pain had interfered “in the past 24 hours” with different functional aspects. This score was the average of the 7 scores, and the total score was calculated as a composite score.

Cognition related to pain: Catastrophizing one’s perception of pain was measured using the Pain Catastrophizing Scale (PCS). This scale has been shown to have high internal consistency (Cronbach α range .67-.87) [42]. The PCS comprises 13 items that evaluate the degree of catastrophizing cognition about pain. The responses are recorded on a 5-point Likert scale, where 0=not at all to 4=all the time. The total PCS scores ranged from 0 to 52, and the clinical cutoff value for the score was over 30 [42,43].

Disability: The degree of life disability due to pain was measured using PDAS. It is composed of 3 factors and supported by a high level of internal consistency (Cronbach α range .87-.95). It consists of 20 items on a 4-point Likert scale and is evaluated from 0 to 60 points, with a higher score indicating a higher degree of daily disability [44]. The clinical cutoff for PDAS is 10 points.

Depression and anxiety: Depressive symptoms were assessed with Beck’s Depression Inventory II and Patient Health Questionnaire-9 items (PHQ-9). BDI-II has an internal consistency of approximately .90, and the test-retest reliability ranges from .73 to .96. It consists of 21 items with 4 response statements designed to assess the severity of current symptoms of depressive disorders. The total scores on the measure range from 0 to 63. Scores below 10 are regarded as reflecting “minimal or no” depression, whereas score ranges of 10-18, 19-29, and 30-63 reflect “mild to moderate,” “moderate to severe,” and “severe” depression, respectively [45,46]. PHQ-9 has diagnostic validity (for the diagnosis of any one or more PHQ disorders, κ=0.65; overall accuracy, 85%; sensitivity, 75%; specificity, 90%). It consists of 9 items scored on a 4-point Likert scale (0=not at all, 1=on several days, 2=half or more of days, and 3=almost daily). The minimum score is 0 and the maximum score is 27 (0-4, 5-9, 10-14, 15-19, and 20-27, indicating no, mild, moderate, moderate to severe, and severe symptoms, respectively) [47]. The PHQ cutoff score for clinically significant depressive symptoms is 10. Anxiety was measured on the Generalized Anxiety Disorder (GAD-7) scale, which has been shown to have reliability, criterion, construct, factorial, and procedural validity. Cutoff points that optimized sensitivity (89%) and specificity (82%) were identified. The scale has 7 items that assess the severity of GAD in the previous 2 weeks on a 4-point Likert scale (0=not at all, 1=one episode, 2=on half or more days, and 3=almost daily). The minimum score is 0 and the maximum score is 21 (0-4, 5-9, 10-14, and 15-21 indicating no, mild, moderate, and severe symptoms, respectively). The cutoff score for clinically significant symptoms of anxiety is 10 [48].

Health-related quality of life: European quality of life 5-dimensions 5-level (EQ-5D-5L) is a widely applied, valid, and reliable measure of quality of life. Its reliability was shown by Cronbach alpha (.70) [49]. EQ-5D-5L consists of 5 items related to mobility, self-care, common activities, pain/discomfort, and anxiety/depression. Patients answer each item on a scale of 1 to 5 (good to severe), and based on the score, the utility value, 0 to 1 (death to in good health), is calculated from the conversion device, which is used for medical economic evaluation [50,51].

#### Medical Economic Evaluation

All enrolled patients were asked to collect and submit receipts of their medical and drug expenses for chronic pain during this study (for 4 months). The incremental cost-effectiveness ratio (ICER) for the comparators vCBT versus TAU was estimated based on prepost evaluation (from baseline to 16 weeks), differences in medical costs, and effects of the condition. To estimate the cost utility of the intervention, quality-adjusted life years (QALYs) were calculated using the utility score of EQ-5D. To calculate the ICER, QALY values were calculated by multiplying one life year by the quality of life score. Quality of life scores were measured using the EQ-5D. We could not use quality of life scores 1 year (52 weeks) after the start of the intervention since the follow-up study had not been done. Therefore, we assumed that quality of life scores at week 16 could return to baseline at 1 year (week 52) as the minimum effect case. Conversely, we assumed that quality of life scores at week 16 could be the same as baseline at 1 year (week 52) as the maximum effect case.

#### Sample Size

In this study, we assumed that the difference in the amount of change in the NRS was 1.67 and the standard deviation was 1.8 and set the detection power to 80% and bilateral significance level to 5% in the two-sided *t* test. As a result, the required number of subjects per group was estimated to be almost 20. In the main analysis, analysis of covariance (ANCOVA) with the allocation factor as the covariate was used, and the detection power was calculated to be 82%. As this study is a pilot study, the number of cases was determined based on its feasibility.

#### Data Management

All data were properly managed by the submitting the case report form to the Clinical Research Data Center. In this center, researchers entered all the data using an access-log-restricted data system, which could be verified, and they created data sets. Independent data monitoring committees were regularly held and they performed risk-based monitoring. After the intervention was finished, the responsible doctors confirmed their data sets and locked the data. Then the locked data were transferred to the Pharmaceutical Statistics Office of the Department of Clinical Trials, Chiba University Hospital.

#### Statistical Analysis

Statistical analysis and reporting of this trial were conducted in accordance with the Consolidated Standards of Reporting Trials guidelines. Baseline variables were compared using Fisher exact test for categorical outcomes and an unpaired two-tailed *t* test for continuous variables. The significance level was set at .05. For the primary analysis of comparing treatment effects, the means of the least squares and their 95% CIs were estimated by ANCOVA with the change in the NRS composite score at week 16. This ANCOVA model took into account the variation caused by treatment effects, and gender and baseline NRS scores (≥6.3 and <6.3) were entered as covariates. As a sensitivity analysis, we showed the transition over time of the NRS scores of each group, confirmed the time course measurement data using the linear mixed effect model, and confirmed that it was not significantly different from the covariance analysis result. All comparisons were planned, and all *P* values were two-sided. *P* values <.05 were considered statistically significant. No special complementary processing by statistical methods was performed for the missing values. However, if necessary, complementary analysis using Mixed Model Repeated Measure was carried out for exploratory analysis. This analysis plan was created before the trial was started, and the protocol was approved. All statistical analyses were performed using SAS V.9.4. (SAS Institute, Cary).

## Results

### Participants

**Figure 1:**
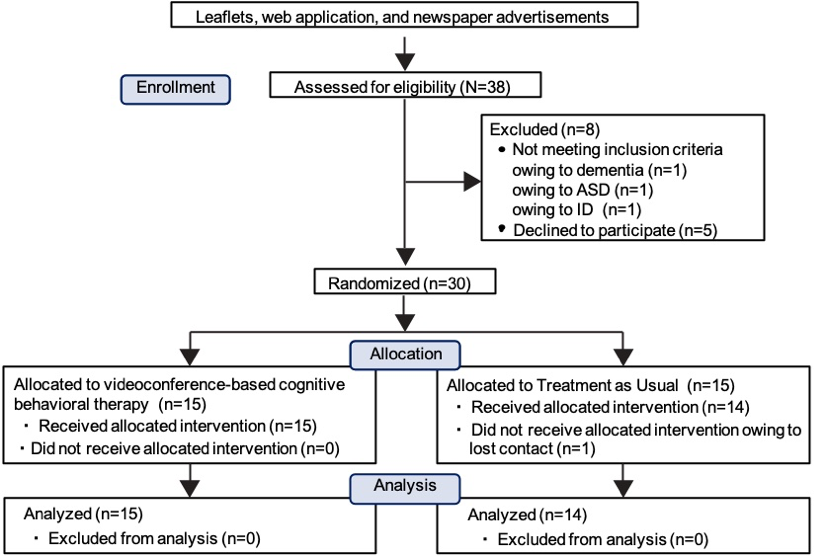
Flowchart of participant selection in the trial. ASD: autism spectrum disorder; ID: intellectual disability.

Figure 1 depicts the participant enrollment for this study. The recruitment process resulted in a total of 38 participants. Three participants were excluded owing to dementia (n=1), autism spectrum disorder (n=1), suspected intellectual disability (n=1), and 5 declined to participate. In the eligibility assessment, 30 patients were eventually enrolled and randomized.

### Patients’ Demographic and Clinical Characteristics

A total of 93% of the patients (14/15) completed all 16 sessions of the vCBT program and participated in the intervention throughout its duration; 2 patients missed 1 session each due to adverse events (see below). No patients in the vCBT group were excluded from the analysis. One patient in the TAU group did not report receiving regular medical treatment at week 8 and was excluded from the analysis. Finally, 15 patients in the vCBT and 14 patients in the TAU group were analyzed.

### Adverse Events

In this study, 4 adverse events were reported by 4 different patients in the vCBT group. The first patient was hospitalized owing to worsening Behcet disease and declined to participate in the study for the fourth session. The second patient had sudden difficulty in opening his eyes due to medically unexplained eyelid pain and declined to participate in the study for the fifth session. The third patient had common cold. The fourth patient had temporomandibular joint disorders.

Table 2 shows the demographic data of the patients. There were no significant differences between vCBT and TAU in age, gender, length of education, employment status, and number of families living together (*P*=.24, *P*>.99, *P=*.73, *P=*.70, *P=*.73; respectively). Duration of illness in vCBT (mean 11.03 [SD 12.64] years) was significantly longer than that in TAU (mean 7.56 [SD 5.84] years). In both groups, more than 65% (19/29) of the patients were women. All patients in the vCBT group had an education period of 12 years or more (high school graduation or above), and TAU group had also 12 years or more except for one patient (9 years). There was no significant difference between the two groups. At baseline, nearly 70% (20/29) of patients were not working (vCBT 11/15, 55% vs TAU 9/14, 45%). Patients were living with at least one family, and only 1 patient receiving vCBT lived alone. The most reported site of chronic pain was lower back pain. Many patients had orthopedic pain, while others had oral pain such as tongue pain, toothache, and general pain such as rheumatism and fibromyalgia.

### Primary Outcomes

Table 3 shows the adjusted mean reductions of NRS in vCBT and TAU at 16 weeks (primary outcome) and at 8 weeks (secondary outcome) from the baseline. No significant difference was found between the 2 groups in terms of changes in composite NRS scores at week 16 from baseline (*P=*.36). Table 4 shows the raw data on the means and standard deviations of NRS scores in vCBT and TAU at 16 weeks and 8 weeks.

**Table 3:** Results of the efficacy on pain intensity in videoconference-based cognitive behavioral therapy versus treatment as usual.

Variable	At 8 weeks					At 16 weeks			
	Estimated amount of change	SE	*P* value	95% CI	Estimated amount of change		SE	*P* value	95% CI
Numerical rating scale (composite)	–0.28	0.49	.57	–1.29 to 0.72	–0.46		0.49	.36	–1.47 to 0.55
Numerical rating scale (max)	–0.13	0.54	.82	–1.24 to 0.99	–0.08		0.55	.88	–1.21 to 1.05
Numerical rating scale (min)	–0.29	0.54	.60	–1.41 to 0.83	–1.04		0.55	.07	–2.17 to 0.09
Numerical rating scale (usual)	–0.43	0.60	.47	–1.66 to 0.79	–0.04		0.60	.94	–1.27 to 1.18

**Table 4:** Change in numerical rating scale scores at the first, eighth, and sixteenth week.

Variable	At 1 week						At 8 weeks							At 16 weeks				
	vCBT^a^			TAU^b^			vCBT			TAU			vCBT				TAU	
	n	Mean (SD)	n		Mean (SD)	n		Mean (SD)	n		Mean (SD)	n			Mean (SD)	n		Mean (SD)
NRS^c^ (composite)	15	5.67 (1.62)	14		5.01 (1.37)	13		5.42 (1.99)	14		4.92 (1.27)	13			5.08 (2.33)	14		4.76 (1.78)
NRS (max)	15	7.30 (1.42)	14		7.40 (1.40)	13		7.08 (1.49)	14		7.18 (1.77)	13			6.42 (2.18	13		6.38 (2.11)
NRS (min)	15	4.33 (2.04)	14		3.01 (1.71)	13		4.22 (2.19)	14		3.20 (1.44)	13			3.74 (2.43)	13		3.52 (1.95)
NRS (usual)	15	5.40 (1.77)	14		4.45 (1.45)	13		5.00 (2.58)	14		4.41 (1.07)	13			5.37 (2.20)	14		4.39 (1.85)

^a^vCBT: videoconference-based cognitive behavioral therapy.

^b^TAU: treatment as usual.

^c^NRS: numerical rating scale.

### Secondary Outcomes

Tables 5 and 6 show the results of efficacy on the secondary outcomes. *P* values lesser than .05 were considered statistically significant. No significant difference was found between the 2 groups regarding changes in maximum, minimum, and usual NRS scores at week 16 from the baseline. In addition, there was no significant difference in the changes in all NRS scores at week 8 from baseline.

Comprehensive pain score: The adjusted mean reduction of the total BPI score in vCBT was significantly larger than that of TAU at 16 weeks (–1.43, 95% CI –2.49 to –0.37, *df*=24; *P*=.01). There was no significant difference between the 2 groups in the adjusted mean reduction of BPI pain severity at 16 weeks. The adjusted mean reduction of BPI pain interference in vCBT was significantly larger than that of TAU at 16 weeks (–1.95, 95% CI –3.33 to –0.56, *df*=24; *P*=.008). There was no significant difference between the 2 groups in the adjusted mean reduction of BPI total score, pain severity, and pain interference at 8 weeks.

Disability: The adjusted mean reduction of the PDAS score in vCBT was significantly larger than that of TAU at 16 weeks (–9.42, 95% CI –14.47 to –4.36, *df*=25; *P*=.001). There was no significant difference between the 2 groups in the adjusted mean reduction of PDAS at 8 weeks.

Catastrophizing cognition: The adjusted mean reduction of the PCS score in vCBT was larger than that of TAU at 16 weeks, although the difference was not statistically significant (–6.25, 95% CI –12.89 to 0.38, *df*=25; *P=*.07). There was no significant difference between the 2 groups in the adjusted mean reduction of PCS at 8 weeks.

Depression and anxiety: There was no significant difference between the 2 groups in the adjusted mean reduction of BDI-II, PHQ-9, and GAD-7 at 16 weeks and 8 weeks, respectively.

Health-related quality of life: The adjusted mean change of the EQ-5D-5L index score in vCBT was larger than that of TAU at 16 weeks, although the difference was not statistically significant (0.09, 95% CI –12.89 to 0.38; *P=*.06). There was also no significant difference between the 2 groups in the adjusted mean change of the EQ-5D-5L index score at 8 weeks.

**Table 5:** Results of the efficacy on secondary outcomes in videoconference-based cognitive behavioral therapy versus treatment as usual.

Outcome	At 8 weeks					At 16 weeks			
	Estimate	SE	*P* value	95% CI	Estimate		SE	*P* value	95% CI
BPI-J^a^ (total)	–0.52	0.51	.33	–1.57 to 0.54	–1.43		0.51	.01	–2.49 to –0.37
BPI-J (pain severity)	0.19	0.46	.68	–0.76 to 1.14	–0.38		0.46	.42	–1.33 to 0.57
Pain worst	–0.25	0.56	.66	–1.41 to 0.91	–1.19		0.56	.05	–2.35 to –0.03
Pain least	1.15	0.55	.05	0.02 to 2.27	0.32		0.55	.56	–0.80 to 1.45
Pain average	0.21	0.55	.70	–0.92 to 1.35	–0.41		0.55	.47	–1.55 to 0.73
Pain current	–0.20	0.69	.77	–1.61 to 1.21	–0.11		0.69	.87	–1.52 to 1.30
BPI-J (pain interference)	–0.88	0.67	.20	–2.26 to 0.51	–1.95		0.67	.01	–3.33 to –0.56
PDAS^b^	–2.71	2.45	.28	–7.77 to 2.34	–9.42		2.45	<.001	–14.47 to –4.36
PCS^c^	–4.20	3.22	.20	–10.84 to 2.44	–6.25		3.22	.06	–12.89 to 0.38
BDI-II^d^	2.33	2.63	.38	–3.08 to 7.74	–2.01		2.63	.45	–7.42 to 3.39
PHQ-9^e^	–0.59	1.47	.69	–3.62 to 2.44	–2.15		1.47	.16	–5.18 to 0.89
GAD-7^f^	0.87	1.4	.54	–2.02 to 3.75	–1.28		1.4	.37	–4.16 to 1.61
EQ-5D-5L^g^	–0.03	0.04	.57	–0.11 to 0.06	0.09		0.04	.05	0.00 to 0.18

^a^BPI-J: Brief Pain Inventory-Japanese translation.

^b^PDAS: Pain Disability Assessment Scale.

^c^PCS: Pain Catastrophizing Scale.

^d^BDI-II: Beck’s Depression Inventory II.

^e^PHQ-9: Patient Health Questionnaire-9 items.

^f^GAD-7: Generalized Anxiety Disorder Scale-7 items.

^g^EQ-5D-5L: European quality of life 5-dimensions 5-level.

**Table 6:** Change in the secondary outcomes in the first, eighth, and sixteenth week.

Variable	Preintervention (1st week)				Midintervention (8th week)						Postintervention (16th week)					
	vCBT^a^		TAU^b^		vCBT			TAU			vCBT			TAU		
	n	Mean (SD)	n	Mean (SD)	n	Mean (SD)	n		Mean (SD)	n		Mean (SD)	n		Mean (SD)	
BPI-J^c^ (total)	14	5.42 (1.74)	14	5.32 (1.27)	13	4.89 (1.71)	14		5.24 (1.33)	13		3.98 (1.83)	14		5.30 (1.63)	
BPI-J (pain severity)	15	5.75 (1.35)	14	5.43 (1.70)	13	5.71 (1.71)	14		5.27 (1.02)	13		4.92 (1.84)	14		5.05 (1.36)	
Pain worst	15	8.07 (1.22)	14	8.14 (1.56)	13	7.54 (1.05)	14		7.79 (1.42)	13		6.38 (1.80)	14		7.57 (1.83)	
Pain least	15	3.60 (1.64)	14	2.71 (2.61)	13	4.15 (2.34)	14		2.43 (1.34)	13		3.62 (2.02)	14		2.71 (1.49)	
Pain average	15	5.67 (1.45)	14	5.57 (1.83)	13	5.38 (1.94)	14		5.07 (1.21)	13		4.69 (1.97)	14		5.00 (1.62)	
Pain current	15	5.67 (2.02)	14	5.29 (2.16)	13	5.77 (2.31)	14		5.79 (1.67)	13		5.00 (2.27)	14		4.93 (2.02)	
BPI-J (pain interference)	14	5.18 (2.35)	14	5.27 (1.47)	13	4.42 (2.19)	14		5.22 (1.83)	13		3.44 (2.05)	14		5.44 (1.97)	
PDAS^d^	15	30.47 (10.35)	14	27.14 (9.29)	13	24.00 (8.55)	14		23.93 (8.65)	13		19.15 (10.04)	14		25.79 (12.24)	
PCS^e^	15	28.87 (10.39)	14	34.50 (6.26)	13	24.15 (10.27)	14		32.71 (8.00)	13		21.38 (10.59)	14		32.00 (10.78)	
PHQ-9^f^	15	11.13 (5.64)	14	11.29 (6.22)	13	9.77 (4.95)	14		10.36 (5.17)	13		8.00 (5.15)	14		10.14 (5.80)	
GAD-7^g^	15	6.13 (4.96)	14	8.71 (4.78)	13	6.69 (4.35)	14		7.21 (4.25)	13		4.69 (3.64)	14		7.36 (5.17)	
BDI-II^h^	15	18.67 (7.66)	14	19.64 (8.87)	13	17.38 (10.74)	14		16.64 (10.63)	13		13.54 (8.32)	14		17.14 (10.31)	
EQ-5D-5L^i^	15	0.54 (0.17)	14	0.55 (0.11)	13	0.57 (0.24)	14		0.61 (0.09)	13		0.60 (0.22)	14		0.52 (0.17)	

^a^vCBT: videoconference-based cognitive behavioral therapy.

^b^TAU: treatment as usual.

^c^BPI-J: Brief Pain Inventory-Japanese translation.

^d^PDAS: Pain Disability Assessment Scale.

^e^PCS: Pain Catastrophizing Scale.

^f^PHQ-9: Patient Health Questionnaire-9 items.

^g^GAD-7: Generalized Anxiety Disorder Scale-7 items.

^h^BDI-II: Beck’s Depression Inventory II.

^i^EQ-5D-5L: European quality of life 5-dimensions 5-level.

### Medical Economic Evaluation

**Figure 2:**
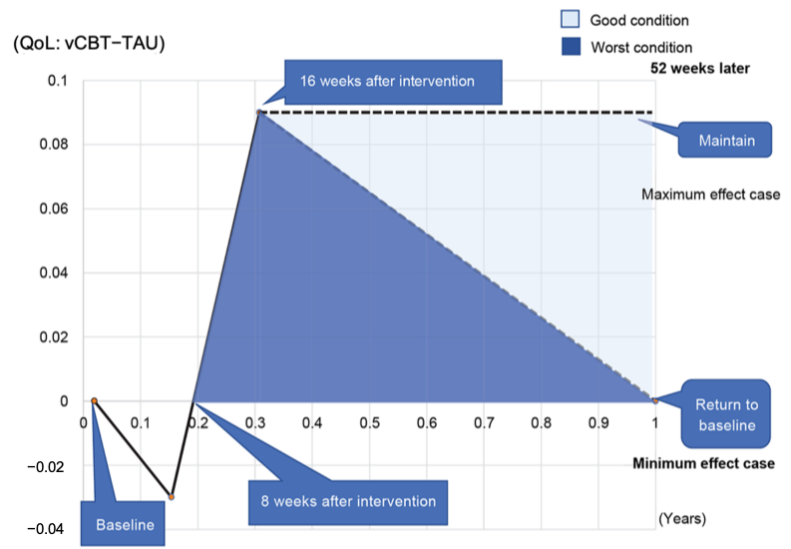
Difference in quality-adjusted life year between both groups at 1 week, 8 weeks, and 16 weeks, and prediction of difference in quality-adjusted life year after 1 year. QALY: quality-adjusted life year; TAU: treatment as usual; vCBT: videoconference-based cognitive behavioral therapy.

Complete data for the direct medical economic evaluation were available for 12 (80%) patients in the vCBT group and 12 (80%) patients in the TAU group with their informed consent. The data consisted of medical expenses covered by public health care insurance, prescription drug prices, and uncovered health care expenses for chronic pain during the intervention period (16 weeks or 4 months). The mean total cost per person for 4 months was 145,846 yen (1 USD=114.05 yen) in vCBT and 169,312 yen in the TAU group, thereby showing no significant difference between the 2 groups (*P=*.73) (Table 7). To calculate the ICER, we extracted incremental effects from EQ-5D and incremental costs from collected receipts of medical and drug expenses. The incremental effect of vCBT on TAU was determined to be 0.033 to 0.064 QALY for 1 year. We adopted 0.033 QALY as the incremental effect as the best case. Regarding the incremental cost, the total cost of 16 sessions of the vCBT program was 96,000 yen per person with 6000 yen per session. Therefore, we adopted 96,000 yen as the incremental cost for vCBT compared with TAU. A cost-benefit analysis based on the data showed that the ICER was almost 2.9 million (yen/QALY=96,000 yen divided by 0.033 QALY). Previous research that used willingness to pay (WTP) to obtain the criterion of the ICER showed that 5.0 million yen (US $48,158) per QALY gained is considered an acceptable threshold in Japan [52]. Therefore, it was suggested that vCBT is more cost-effective than TAU, even though it was a minimum effect case, as the improvement in quality of life returned to the baseline in 1 year (shown in Figure 2).

**Table 7:** Comparison of costs (in yen) for treatment of chronic pain.^a^

Costs			1st month		2nd month		3rd month		4th month		Total amount		*P* value	
**Insurance medical treatment for chronic pain (100% burden), mean (SD)**														.69
	TAU^b^ (n=12)	16,057 (16,232)		15,898 (16,711)		10,681 (14,060)		18,014 (23,755)		60,650 (64,292)				
	vCBT^c^ (n=12)	9309 (8754)		19,182 (24,923)		28,728 (42,755)		17,153 (33,133)		74,372 (98,889)				
**Outpatient prescription amount for chronic pain, mean (SD)**														.99
	TAU (n=12)	13,503 (14,082)		16,373 (17,924)		12,163 (11,756)		19,517 (23,939)		61,556 (58,758)				
	vCBT (n=12)	23,129 (28,480)		8149 (10,933)		15,578 (15,318)		15,078 (14,047)		61,933 (59,961)				
**Private insurance amount for chronic pain, mean (SD)**														.35
	TAU (n=12)	9637 (23,676)		12,049 (29,535)		11,710 (32,480)		13,710 (41,539)		47,106 (132,486)				
	vCBT (n=12)	1817 (4051)		2233 (6249)		3811 (8236)		1680 (3723)		9541 (21,859)				
**Total cost for chronic pain, mean (SD)**														.73
	TAU (n=12)	39,197 (39,425)		44,319 (46,594)		34,554 (50,316)		51,241 (63,891)		169,312 (195,072)				
	vCBT (n=12)	34,255 (29,983)		29,564 (23,755)		48,116 (45,076)		33,910 (35,109)		145,846 (123,008)				

^a^1 USD=114.05 yen.

^b^TAU: treatment as usual.

^c^vCBT: videoconference-based cognitive behavioral therapy.

## Discussion

To our knowledge, this study is the first RCT to evaluate the effectiveness of vCBT for chronic pain. In addition, we performed a cost-effectiveness analysis of the CBT program by using ICER. Although composite pain intensity by the NRS did not change, vCBT significantly improved the total BPI score, especially pain interference and disability in daily life. Furthermore, in the medical economic evaluation, although our results showed no statistically significant difference, it has been suggested that vCBT may be more cost-effective than TAU for various reasons that we discuss in the following sections.

The latest systematic review of 75 RCTs about the effectiveness of face-to-face CBT has shown that CBT versus TAU at the treatment end for pain, disability, and distress showed a small effect size, and versus active treatment showed few effects (n=9401) [9]. In general, internet-based CBT is delivered through a computer application for administering self-management programs without a therapist. According to a systematic review of internet-based CBT for depression, anxiety disorder, and other functional disorders, including chronic pain, the CBT for chronic pain showed small to medium effect size from 10 RCT studies with low levels of evidence. Conversely, internet-based CBT for depression, social anxiety disorder, and panic disorder was classified as established, that is, they met the highest level of evidence criteria [11]. A systematic review of internet-based CBT for lower back pain reported high effectiveness for pain intensity and disability in 9 RCT studies (n=1796) [53]. Although there are several studies on internet-based CBT, there are only a few RCTs on vCBT, and almost all of them focus on self-care by patients. Only a pilot study of hematopoietic stem cell transplantation has reported on the feasibility and improved self-efficacy of vCBT [26].

As reported by multiple internet-based CBT studies, use of self-care programs or apps, treatments in which specialists check progress every other week, or telephone sessions have been demonstrated to be effective. Such internet-based CBT does not require multiple therapists, and interventions can be conducted for many patients concurrently. However, there is insufficient evidence for the effectiveness of the treatment. Chronic pain is unique to each individual and therefore, there is a need to consider stepped care for chronic pain, which is a system of delivering an evidence-based staged approach and provides treatment that matches individual needs and status. Ideally, it is desirable for patients to receive internet-based CBT in the early stage as first aid, such as through a self-care smartphone app. Moreover, patients who do not respond well to first aid should receive vCBT as the second aid.

vCBT is intended for the treatment of higher levels of chronic pain. We need to examine treatments for several levels of pain for treating complex and intractable chronic pain. This was the first RCT on vCBT showing significant improvement in interference (distress) and disability. Our primary outcome, pain intensity as measured using NRS, was not significantly different; however, we adopted the BPI total score as a comprehensive pain score, which showed significant results. The reason for the NRS result could be that TAU also improved pain intensity. According to the BPI total score, pain interference appeared to greatly improve, while pain severity only showed a slight change, which comprehensively improved the total score. We hypothesized that patients who received CBT increased their previously avoided daily movement by doing the CBT-related homework and confronting their pain, thus maintaining their pain intensity. Conversely, it was possible for patients with TAU to avoid several behaviors owing to pain; as a result, their pain interference score worsened gradually. Considering this development, a composite score such as a BPI total score should be adopted for assessing the effectiveness of treatment for chronic pain. A recent consensus recommended the use of comprehensive indicators such as a BPI [54]. In particular, the composite score of BPI is the right choice because it comprises 2 domains, pain severity and interference due to pain, which are measured as outcomes in all chronic pain clinical trials. Few studies have set the BPI total score as primary [23] and there might be a need to set a comprehensive scale, instead of a single rating scale, as the primary outcome in future research.

We expected improvement of cognition-related pain-related catastrophic thoughts, anxiety, and depressive symptoms, because a previous review had shown small-to-moderate effect size of catastrophic thinking and mood [9]. Moreover, one of the new additional sessions of our protocol focused on emotions related to pain (session 10). Contrary to expectations, there was no significant difference between PCS and mood. However, if the sample size is a little large, it may show a positive result as the PCS score of vCBT improves compared to TAU (shown in Table 6). Considering the previous review, it is not easy to improve mood by CBT for chronic pain [55]. Our new protocol also did not treat their anxiety and depression.

We thus added a new session to address emotions such as anxiety and depression. However, in this session, many patients could not recognize the emotions related to pain. They thought of their pain and emotions as separate and did not acknowledge emotions that accompanied the pain memory or emotions that accompanied the moment pain was felt. An RCT found that emotion-focused exposure intervention (10-15 sessions) compared to normal CBT for chronic pain is effective for catastrophic cognition and depression, in addition to other outcomes [56]. The research concluded that the emotion-focused approach is an effective option. We had a single emotion-focused session, which was too little time for patients to learn how to treat their emotions.

In medical economic evaluation, a cost-benefit analysis showed that ICER was almost 2.9 million (yen/QALY), which was lesser than Japan's WTP of 5 million yen. Furthermore, it was also shown to be lesser than the British WTP of £23,000 (approximately 3 million yen) and the United States WTP of $62,000 (approximately 6.7 million yen) [51]. At postintervention (16 weeks), the total cost of each group was not significantly different; however, the cost of vCBT did not change even though TAU increased gradually. In particular, the decrease in outpatient prescriptions of vCBT increased TAU costs, which may have affected the overall costs. We understand that the results of economic assessments are somewhat uncertain for a variety of reasons. Since this was a pilot study, the number of verifications was small. In addition, since no follow-up data were taken, the cost-effectiveness results were derived from assumptions of the sustainability of therapeutic efficacy. This uncertainty should be kept in mind if clinicians and policymakers set treatment guidelines or offer this program in a clinical setting.

This study has several limitations. First, the sample size was relatively small. In addition, this study was performed as a single-center study at our hospital. In the near future, large-scale multicenter trials are necessary. Second, further studies targeting patients with specific types of chronic pain will be required to examine the effectiveness of this vCBT program. Third, because we did not use a psychological placebo group as a control condition, we were unable to control nonspecific factors and unravel the concrete effects of the vCBT program. Finally, the lack of follow-up data limited the generalizability of the conclusions. Long-term follow-up studies should be conducted in the future.

Despite the problems in this study, vCBT has the possibility of usability and effectiveness for patients with chronic pain. During COVID-19, in particular, vCBT may provide safety and alleviate the patient’s stress through face-to-face counseling. In the future, we may study other pandemics and help patients to decrease their anxiety and symptoms by using internet-based CBT.

In summary, videoconference-based integrated CBT for chronic pain could improve pain interference and disability in daily life, and as a result, could even relieve comprehensive pain. Therefore, this program can be a valuable addition to regular medical care. Further research is needed to examine augmentation strategies, including an examination of the components of CBT that are best suited for different types of pain. Furthermore, while this study indicated the cost-effectiveness of this treatment within a small sample, this needs to be verified with a larger sample size.

## Appendix

Multimedia Appendix 1 CONSORT-eHEALTH checklist (V 1.6.1).

## Abbreviations

ANCOVA: analysis of covariance
BPI: Brief Pain Inventory
CBT: cognitive behavioral therapy
DSM-5: Diagnostic and Statistical Manual of Mental Disorders, Fifth Edition
EQ-5D-5L: European quality of life 5-dimensions 5-level
GAD-7: Generalized Anxiety Disorder scale-7 items
ICER: incremental cost-effectiveness ratio
NRS: numerical rating scale
PCS: Pain Catastrophizing Scale
PDAS: Pain Disability Assessment Scale
PHQ-9: Patient Health Questionnaire-9 items
QALY: quality-adjusted life year
RCT: randomized controlled trial
TAU: treatment as usual
vCBT: videoconference-based cognitive behavioral therapy
WTP: willingness to pay
