# Supplementary material for: Clinical Effectiveness and Cost-effectiveness of Videoconference-Based Integrated Cognitive Behavioral Therapy for Chronic Pain: Randomized Controlled Trial
Source: J Med Internet Res. 2021 Nov 22;23(11):e30690. doi: 10.2196/30690 (PMC8663446; doi:10.2196/30690)
Supplement: Multimedia Appendix 1 [file jmir_v23i11e30690_app1.pdf]

CONSORT-EHEALTH Checklist V1.6.2 Report

Manuscript Number 30690

(based on CONSORT-EHEALTH V1.6), available at [http://tinyurl.com/consort-ehealth-v1-6].

Date completed

7/16/2021 7:27:31

by

Kayoko Taguchi

Clinical and Cost-effectiveness of Videoconference-based Integrated Cognitive Behavioral Therapy for Chronic Pain: A Randomized Controlled Trial

TITLE

1a-i) Identify the mode of delivery in the title

We use " Videoconference-based " as the mode of delivery. It is means web- based.

1a-ii) Non-web-based components or important co-interventions in title

1a-iii) Primary condition or target group in the title

If case that target is children, we think its need subitem 1a- iii , but our study for adults so it's need not subitem, we thought.

ABSTRACT

1b-i) Key features/functionalities/components of the intervention and comparator in the METHODS section of the ABSTRACT

We showed mention key features of the intervention as "integrated cognitive behavioral program with new components (attention-shift, memory work, video feedback, and image training) delivered via videoconferencing" in OBJECTIVE.

1b-ii) Level of human involvement in the METHODS section of the ABSTRACT

We conducted a randomized controlled trial for chronic pain to compare 16 weekly videoconference-based web cognitive behavioral therapy sessions "provided by therapist" with treatment as usual.

1b-iii) Open vs. closed, web-based (self-assessment) vs. face-to-face assessments in the METHODS section of the ABSTRACT

"This study was unblind and participants were recruited face to face in the outpatient department."

1b-iv) RESULTS section in abstract must contain use data

"30 patients were eventually randomized and enrolled; finally, 15 patients in the vCBT and 14 patients in the TAU group were analyzed. Although no significant difference was found between the two groups in terms of changes in pain intensity by Numerical Rating Scale scores at week 16 from baseline ($p = 0.357$), there was a significant improvement in the comprehensive evaluation of pain by total score of Brief Pain Inventory (-1.43 [95% CI -2.49 to -0.37], $df = 24$, $p = 0.010$). Further, significant improvement was seen in pain interference by BPI (-9.42 [95% CI -14.47 to -4.36], $df = 25$, $p = 0.001$) and disability by the Pain Disability Assessment Scale (-1.95 [95% CI -3.33 to -0.56], $df = 24$, $p = 0.008$) compared with treatment as usual. As for Medical Economic Evaluation, the incremental cost-effectiveness ratio for one year was estimated at 2.9 million yen per quality-adjusted life year gained (JPY/QALY =96,000; JPY divided by 0.033 QALY)."

1b-v) CONCLUSIONS/DISCUSSION in abstract for negative trials

Our results suggested integrated cognitive behavioral therapy delivered by videoconferencing on the regular medical care may be reduced in pain interference "although pain intensity was not changed".

INTRODUCTION

2a-i) Problem and the type of system/solution

While such remote treatment is known by several names, such as internet-delivered CBT (ICBT), web-based CBT, telemedicine, etc., these are all strictly different interventions. Web-based CBT and ICBT were used synonymously in a study in which patients performed CBT on their own as self-help training or received regular therapist feedback that was not face-to-face [19,20]. Moreover, many past studies involved treatment programs delivered via the internet or through smart phone applications, with the aim of self-management by patients (no intervention by the therapist) [54,21,22].
Only a few studies have examined the effectiveness of videoconference CBT, which is face-to-face CBT using a videoconference system, and not general ICBT, for chronic pain management [23,24,25].

2a-ii) Scientific background, rationale: What is known about the (type of) system

The latest review on the effect of CBT verified by randomized controlled trials (RCTs) has added 41 new studies to the existing 34 studies, thus creating a larger pool of 75 verified RCTs. Comparisons of CBT with active controls showed a slight benefit in terms of pain intensity, disability, and distress immediately after treatment [10]. It was also observed that there were small merits of each of the three outcomes as compared to no treatment. At follow-up, pain, disability, distress, and other variables were maintained in comparison with no treatment, but there is a lack of rigorous studies that involve active controls [10]. In summary, there is evidence of efficacy, although the effect size of CBT for chronic pain is small and insufficient.
Only a few studies have examined the effectiveness of videoconference CBT, which is face-to-face CBT using a videoconference system, and not general ICBT, for chronic pain management [23,24,25].

Does your paper address CONSORT subitem 2b?

The aim of this study was to verify the cost- and clinical effectiveness of a new integrated CBT program for chronic pain, delivered through videoconferencing (vCBT), compared with treatment as usual (TAU), for pain intensity, pain interference, disability, pain-related catastrophizing cognition, depression, anxiety, quality of life, and cost utility.

METHODS

3a) CONSORT: Description of trial design (such as parallel, factorial) including allocation ratio

This study was designed as a prospective randomized open-labeled pilot trial comparing vCBT for the intervention group to treatment as usual (TAU) for the control group at the academic outpatient clinic of the Cognitive Behavioral Therapy Center at Chiba University Hospital.

3b) CONSORT: Important changes to methods after trial commencement (such as eligibility criteria), with reasons

There were not important change in our methods because our intervention was web based CBT which face to face therapy and we used existing videoconference system.

3b-i) Bug fixes, Downtimes, Content Changes

4a) CONSORT: Eligibility criteria for participants

Eligibility Procedure for Participation and Diagnosis
Written informed consent was obtained from all patients after they were fully briefed on the procedure. Following this, a screening eligibility assessment for inclusion and exclusion criteria was performed. Participants were asked to record their pain intensity rated by the Numerical Rating Scale (NRS) daily for a week. Inclusion criteria were as follows: (1) fulfillment of the criteria of somatic symptom disorder, with predominant pain according to the DSM-5, (2) age ranging from 18 to 75 years to avoid the risks from cognitive decline; (3) not showing moderate to remarkable improvement despite receiving sufficient pharmacotherapy for more than eight weeks, or due to poor tolerability; and (4) appropriate mental and physical conditions to maintain therapy. In the case of patients suffering from depression or other anxiety conditions, they met the criteria to take part in the study if their pain was the primary impairment. The exclusion criteria were: (1) comorbidity of serious mental disorders such as neurocognitive disorder, psychotic disorder, bipolar disorder, or substance-related disorder based on the criteria in DSM-5; (2) major pain caused by cancer; (3) if their pain did not interfere with their daily life (PDAS: Pain Disability Assessment Scale score of 9 or less); (4) mental retardation, neurocognitive disorders (dementia), and autism spectrum disorder; and (5) litigation or compensation concerning pain symptoms. In this study, patients were required to be able to use a videoconferencing system at home. In case of patients who did not have an internet connection in their houses, we rented tablet computers and mobile Wi-Fi devices for them.

4a-i) Computer / Internet literacy

4a-ii) Open vs. closed, web-based vs. face-to-face assessments:

This study was designed as a prospective randomized "open-labeled" pilot trial comparing vCBT for the intervention group to treatment as usual (TAU) for the control group at the academic outpatient clinic of the Cognitive Behavioral Therapy Center at Chiba University Hospital.
"Our developed protocol which face-to-face CBT sessions provided by videoconference system (Web-based CBT)", has been shown to improve catastrophic cognition, disability, and mood [29].

4a-iii) Information giving during recruitment

All recruitment materials referred patients to our study website, which explained the study in detail. All participants who gave their permission to be enrolled in the study were required to continue treatment with their general practitioners as TAU. Patients who were interested in the study could inquire about the details via e-mail. This mail was also used as an application form to ask patients to record their age, sex, condition of chronic pain, contact information, and so forth.

4b) CONSORT: Settings and locations where the data were collected

All data was properly managed by the submitting case report form to the Clinical Research Data Center. In this center, researchers entered all data using an access-log-restricted data system, which could be verified and created datasets. Independent data monitoring committees were regularly held and performed risk-based monitoring. After all intervention was finished, the responsible doctors confirmed their datasets and locked the data. Then the locked data were transferred to the Pharmaceutical Statistics Office of the Department of Clinical Trials, Chiba University Hospital.

4b-i) Report if outcomes were (self-)assessed through online questionnaires

Our study outcome were self assessed by patients and was reported by mail.

4b-ii) Report how institutional affiliations are displayed

5) CONSORT: Describe the interventions for each group with sufficient details to allow replication, including how and when they were actually administered

**5-i) Mention names, credential, affiliations of the developers, sponsors, and owners**		
Our intervention is CBT program provided by web system, so there were not sponsors except grant.		
**5-ii) Describe the history/development process**		
The CBT program we adopted is an integrated CBT program that is longer than conventional interventions and consists of several new sessions not used in traditional CBT protocols. Our developed protocol which face-to-face CBT sessions provided by videoconference system (Web-based CBT), has been shown to improve catastrophic cognition, disability, and mood [29].<br>Conventional CBT programs for chronic pain often comprise 8–12 intervention sessions. In almost all programs, psychoeducation for pain, case formulation for understanding cognitive behavioral models of chronic pain, relaxation exercises such as breathing, and cognitive reconstruction, among others, were included [30]. Each of the 16 sessions lasted 50 minutes. We added four new sessions: tactile attention-shift training (session 4), memory work based on peak-end rule (session 10), sharpening behavioral image training (session 11), and video feedback (session 12) to the conventional CBT program (shown in Table 1).		
**5-iii) Revisions and updating**		
The web based CBT program did not change throughout this trial.		
**5-iv) Quality assurance methods**		
As quality control, therapists recorded CBT session during the intervention period and maintained equality of intervention throughout supervision by responsible doctor.		
**5-v) Ensure replicability by publishing the source code, and/or providing screenshots/screen-capture video, and/or providing flowcharts of the algorithms used**		
Our intervention was CBT sessions face to face using videoconference system, so there is not source code etc ..		
**5-vi) Digital preservation**		
Our study did not use application.		
**5-vii) Access**		
Our study did not use application.		
**5-viii) Mode of delivery, features/functionalities/components of the intervention and comparator, and the theoretical framework**		
System Safety<br>In this study, we adopted the ISO 27001-certified Cisco WebEx as the internet conference system. Countermeasures against unauthorized access, information leakage, etc., were taken, and safety problems were cleared.		
**5-ix) Describe use parameters**		
While participating in this study, no patient was permitted to seek any new treatment other than those that their primary care doctor ordered. In addition to regular medical care, those allocated to the vCBT group received weekly 50-minute sessions over 16 weeks of integrated CBT program using real-time internet videoconferencing.		
**5-x) Clarify the level of human involvement**		
All sessions were provided by therapist.		
**5-xi) Report any prompts/reminders used**		
The patient received an invitation email, attended a video conference, and attended a CBT session.		
**5-xii) Describe any co-interventions (incl. training/support)**		
There were not co- interventions.		
**6a) CONSORT: Completely defined pre-specified primary and secondary outcome measures, including how and when they were assessed**		
Primary Outcome Measures<br>Pain intensity: The primary outcome was the change from baseline to week 16, as indicated by the NRS score. The NRS is a self-rated questionnaire that measures pain intensity on a scale of 0–10, where 0 = “nothing” and 10 = “severe.” Patients were made to keep a daily pain diary. They recorded (1) maximum pain throughout the day, (2) minimum pain, (3) usual pain, and calculated the weekly average for pain on the day of the session (each NRS score = sum total of 1-week NRS score / 7). Numerical values obtained by averaging the values in (1), (2), and (3) are taken as the main evaluation items comprising the composite value of NRS. The measurement has been shown to be reliable and valid [39].<br>Secondary Outcome Measures<br>All secondary outcomes except the NRS were measured at 8 weeks and 16 weeks from the baseline.<br>Pain intensity: The secondary outcome was change in pain intensity (maximum, minimum, usual score) from baseline to week eight by the NRS.<br>Comprehensive pain score: Comprehensive pain was assessed with the Japanese translation of the Brief Pain Inventory (BPI) [40]. BPI is composed of two factors: pain severity and pain interference. The scale has a high reliability (coefficient alpha greater than 0.80) and established validity. Pain severity on the BPI comprises four items (worst, least, average, and current). They are assessed as 0 = “nothing” and 10 = “severe” with higher scores representing worse pain. Pain severity was calculated as the average of the four scores. Pain interference of BPI is a seven-item measure designed to assess pain interference by sleep, mood, social relations, and enjoyment of life. On an 11-point scale (0 = “does not interfere,” 10 = “completely interferes”), patients indicated how much pain had interfered “in the past 24 hours” with different functional aspects. This score was the average of the seven scores, and the total score was calculated as a composite score.<br>Cognition related to pain: Catastrophizing one’s perception of pain was measured using the Pain Catastrophizing Scale (PCS). The scale has been shown to have high internal consistency (Cronbach’s α range: 0.67 to 0.87) [41]. The PCS comprises 13 items that evaluate the degree of catastrophizing cognition about pain. The responses are recorded on a 5-point Likert scale, where 0 = “not at all” to 4 = “all the time.” The total PCS scores range from 0 to 52, and the clinical cutoff value for the score is over 30 [41,42].<br>Disability: The degree of life disability due to pain was measured using the Pain Disability Assessment Scale (PDAS). It is composed of three factors and supported by a high level of internal consistency (Cronbach’s α range: 0.87 to 0.95). It consists of 20 items on a 4-point Likert scale and is evaluated from 0 to 60 points, with a higher score indicating a higher degree of daily disability [43]. The clinical cutoff for PDAS is 10 points.<br>Depression and anxiety: Depressive symptoms were assessed with Beck’s Depression Inventory II (BDI-II) and Patient Health Questionnaire-9 (PHQ-9). BDI-II has an internal consistency of approximately 0.9, and the test-retest reliability ranges from 0.73 to 0.96. It consists of 21 items with four response statements designed to assess the severity of current symptoms of depressive disorders. The total scores on the measure range from 0 to 63. Scores below 10 are regarded as reflecting “minimal or no” depression, whereas score ranges of 10 to 18, 19 to 29, and 30 to 63 reflect “mild to moderate,” “moderate to severe,” and “severe” depression, respectively [44,45]. PHQ-9 has diagnostic validity (for the diagnosis of any one or more PHQ disorders, kappa = 0.65; overall accuracy, 85%; sensitivity, 75%; specificity, 90%). It consists of nine items scored on a 4-point Likert scale (0 = “not at all,” 1 = “on several days,” 2 = “half or more of days,” and 3 = “almost daily”). The minimum score is 0 and the maximum score is 27 (0–4, 5–9, 10–14, 15–19, and 20–27, indicating “no,” “mild,” “moderate,” “moderate to severe,” and “severe” symptoms, respectively) [46]. The PHQ cutoff score for clinically significant depressive symptoms is 10. Anxiety was measured on the Generalized Anxiety Disorder Scale (GAD-7), which has been shown to have reliability, and criterion, construct, factorial, and procedural validity. Cutoff points that optimized sensitivity (89%) and specificity (82%) were identified. The scale has seven items that assess the severity of GAD in the previous two weeks on a 4-point Likert scale (0 = “not at all,” 1 = “one episode,” 2 = “on half or more days,” and 3 = “almost daily”). The minimum score is 0 and the maximum score is 21 (0–4, 5–9, 10–14, and 15–21 indicating “no,” “mild,” “moderate,” and “severe” symptoms, respectively). The cutoff score for clinically significant symptoms of anxiety is 10 [47].<br>Health-related quality of life: EuroQOL 5-dimensions 5-level (EQ-5D-5L) is a widely applied, valid, and reliable measure of quality of life. Its reliability was shown by Cronbach's alpha (0.70) [48].EQ-5D-5L consists of five items related to mobility, self-care, common activities, pain/discomfort, and anxiety/depression. Patients answer each item on a scale of 1 to 5 (good to severe), and based on the score, the utility value, 0 to 1 (death to in good health), is calculated from the conversion device, which is used for medical economic evaluation [49,50].		
**6a-i) Online questionnaires: describe if they were validated for online use and apply CHERRIES items to describe how the questionnaires were designed/deployed**		
We did not use online questionnaires.		
**6a-ii) Describe whether and how “use” (including intensity of use/dosage) was defined/measured/monitored**		
Our intervention was face to face CBT session using videoconference system, so it is not applicable.		
**6a-iii) Describe whether, how, and when qualitative feedback from participants was obtained**		
Our intervention was face to face CBT session using videoconference system, so it is not applicable.		
**6b) CONSORT: Any changes to trial outcomes after the trial commenced, with reasons**		
All data was properly managed by the submitting case report form to the Clinical Research Data Center. In this center, researchers entered all data using an access-log-restricted data system, which could be verified and created datasets. Independent data monitoring committees were regularly held and performed risk-based monitoring. After all intervention was finished, the responsible doctors confirmed their datasets and locked the data. Then the locked data were transferred to the Pharmaceutical Statistics Office of the Department of Clinical Trials, Chiba University Hospital.		
**7a) CONSORT: How sample size was determined**		
**7a-i) Describe whether and how expected attrition was taken into account when calculating the sample size**		
"Sample size<br>In this study, we assumed that the difference in the amount of change in the NRS was 1.67 and the standard deviation was 1.8, and set the detection power to 80% and bilateral significance level to 5% in the t-test. As a result, the required number of subjects per group was estimated to be almost 20. In the main analysis, analysis of covariance (ANCOVA) with the allocation factor as the covariate was used, and the detection power was calculated to be 82%. As this study is a pilot study, the number of cases was determined based on its feasibility.<br>2		
**7b) CONSORT: When applicable, explanation of any interim analyses and stopping guidelines**		

Primary Outcome Measures
Pain intensity: The primary outcome was the change from baseline to week 16, as indicated by the NRS score. The NRS is a self-rated questionnaire that measures pain intensity on a scale of 0–10, where 0 = “nothing” and 10 = “severe.” Patients were made to keep a daily pain diary. They recorded (1) maximum pain throughout the day, (2) minimum pain, (3) usual pain, and calculated the weekly average for pain on the day of the session (each NRS score = sum total of 1-week NRS score / 7). Numerical values obtained by averaging the values in (1), (2), and (3) are taken as the main evaluation items comprising the composite value of NRS. The measurement has been shown to be reliable and valid [39].
Secondary Outcome Measures
All secondary outcomes except the NRS were measured at 8 weeks and 16 weeks from the baseline.
Pain intensity: The secondary outcome was change in pain intensity (maximum, minimum, usual score) from baseline to week eight by the NRS.
Comprehensive pain score: Comprehensive pain was assessed with the Japanese translation of the Brief Pain Inventory (BPI) [40]. BPI is composed of two factors: pain severity and pain interference. The scale has a high reliability (coefficient alpha greater than 0.80) and established validity. Pain severity on the BPI comprises four items (worst, least, average, and current). They are assessed as 0 = “nothing” and 10 = “severe” with higher scores representing worse pain. Pain severity was calculated as the average of the four scores. Pain interference of BPI is a seven-item measure designed to assess pain interference by sleep, mood, social relations, and enjoyment of life. On an 11-point scale (0 = “does not interfere,” 10 = “completely interferes”), patients indicated how much pain had interfered “in the past 24 hours” with different functional aspects. This score was the average of the seven scores, and the total score was calculated as a composite score.
Cognition related to pain: Catastrophizing one’s perception of pain was measured using the Pain Catastrophizing Scale (PCS). The scale has been shown to have high internal consistency (Cronbach’s α range: 0.67 to 0.87) [41]. The PCS comprises 13 items that evaluate the degree of catastrophizing cognition about pain. The responses are recorded on a 5-point Likert scale, where 0 = “not at all” to 4 = “all the time.” The total PCS scores range from 0 to 52, and the clinical cutoff value for the score is over 30 [41,42].
Disability: The degree of life disability due to pain was measured using the Pain Disability Assessment Scale (PDAS). It is composed of three factors and supported by a high level of internal consistency (Cronbach’s α range: 0.87 to 0.95). It consists of 20 items on a 4-point Likert scale and is evaluated from 0 to 60 points, with a higher score indicating a higher degree of daily disability [43]. The clinical cutoff for PDAS is 10 points.
Depression and anxiety: Depressive symptoms were assessed with Beck’s Depression Inventory II (BDI-II) and Patient Health Questionnaire-9 (PHQ-9). BDI-II has an internal consistency of approximately 0.9, and the test-retest reliability ranges from 0.73 to 0.96. It consists of 21 items with four response statements designed to assess the severity of current symptoms of depressive disorders. The total scores on the measure range from 0 to 63. Scores below 10 are regarded as reflecting “minimal or no” depression, whereas score ranges of 10 to 18, 19 to 29, and 30 to 63 reflect “mild to moderate,” “moderate to severe,” and “severe” depression, respectively [44,45]. PHQ-9 has diagnostic validity (for the diagnosis of any one or more PHQ disorders, kappa = 0.65; overall accuracy, 85%; sensitivity, 75%; specificity, 90%). It consists of nine items scored on a 4-point Likert scale (0 = “not at all,” 1 = “on several days,” 2 = “half or more of days,” and 3 = “almost daily”). The minimum score is 0 and the maximum score is 27 (0–4, 5–9, 10–14, 15–19, and 20–27, indicating “no,” “mild,” “moderate,” “moderate to severe,” and “severe” symptoms, respectively) [46]. The PHQ cutoff score for clinically significant depressive symptoms is 10. Anxiety was measured on the Generalized Anxiety Disorder Scale (GAD-7), which has been shown to have reliability, and criterion, construct, factorial, and procedural validity. Cutoff points that optimized sensitivity (89%) and specificity (82%) were identified. The scale has seven items that assess the severity of GAD in the previous two weeks on a 4-point Likert scale (0 = “not at all,” 1 = “one episode,” 2 = “on half or more days,” and 3 = “almost daily”). The minimum score is 0 and the maximum score is 21 (0–4, 5–9, 10–14, and 15–21 indicating “no,” “mild,” “moderate,” and “severe” symptoms, respectively). The cutoff score for clinically significant symptoms of anxiety is 10 [47].
Health-related quality of life: EuroQOL 5-dimensions 5-level (EQ-5D-5L) is a widely applied, valid, and reliable measure of quality of life. Its reliability was shown by Cronbach's alpha (0.70) [48].EQ-5D-5L consists of five items related to mobility, self-care, common activities, pain/discomfort, and anxiety/depression. Patients answer each item on a scale of 1 to 5 (good to severe), and based on the score, the utility value, 0 to 1 (death to in good health), is calculated from the conversion device, which is used for medical economic evaluation [49,50].

8a) CONSORT: Method used to generate the random allocation sequence

Randomization and Case Registration
"The eligible patients were randomly assigned to either the vCBT group or the TAU group using the minimization method used in clinical trials to ensure a balance in pain intensity score and gender. "Per the allocation adjustment factor, the pain intensity score on NRS was allocated at 6.3 for each group [28].

8b) CONSORT: Type of randomisation; details of any restriction (such as blocking and block size)

Randomization and Case Registration
The eligible patients were randomly assigned to either the vCBT group or the TAU group using the minimization method used in clinical trials to ensure a balance in pain intensity score and gender. "Per the allocation adjustment factor, the pain intensity score on NRS was allocated at 6.3 for each group [28]."

9) CONSORT: Mechanism used to implement the random allocation sequence (such as sequentially numbered containers), describing any steps taken to conceal the sequence until interventions were assigned

The randomization and assign did by the Clinical Research Data Center, and result was informed to researcher by fax.

10) CONSORT: Who generated the random allocation sequence, who enrolled participants, and who assigned participants to interventions

The randomization and assign did by the Clinical Research Data Center, and result was informed to researcher by fax.

11a) CONSORT: Blinding - If done, who was blinded after assignment to interventions (for example, participants, care providers, those assessing outcomes) and how

11a-i) Specify who was blinded, and who wasn’t

Our intervention was face to face CBT session using videoconference system, so it is not applicable.

11a-ii) Discuss e.g., whether participants knew which intervention was the “intervention of interest” and which one was the “comparator”

The purpose of this study is to verify effectivity of Videoconference-based Integrated Cognitive Behavioral Therapy as pilot study, and we informed participants about this. Participants knew that vCBT group was “intervention of interest”,TAU group was “comparator”, as the recruitement stage.

11b) CONSORT: If relevant, description of the similarity of interventions

In our study, this item was not applicable.

12a) CONSORT: Statistical methods used to compare groups for primary and secondary outcomes

Statistical Analysis
Statistical analysis and reporting of this trial were conducted in accordance with the Consolidated Standards of Reporting Trials guidelines." Baseline variables were compared using Fisher’s exact test for categorical outcomes and an unpaired t-test for continuous variables. The significance level was set at 0.05 (two-tailed).
For the primary analysis of comparing treatment effects, the means of the least squares and their 95% CIs were estimated by ANCOVA with the change in the NRS composite score at week 16. This ANCOVA model took into account the variation caused by treatment effects, and gender and baseline NRS scores (≥ 6.3 and < 6.3) were entered as covariates. As a sensitivity analysis, we showed the transition over time of the NRS scores of each group, confirmed the time course measurement data using the linear mixed effect model, and confirmed that it was not significantly different from the covariance analysis result. All comparisons were planned, and all p-values were two-sided. p-values < 0.05 were considered statistically significant. All statistical analyses were performed using SAS V.9.4. (SAS Institute, Cary, NC, USA). "

12a-i) Imputation techniques to deal with attrition / missing values

”No special complementary processing by statistical method is performed for missing values. However, if necessary, complementary analysis using MMRM (Mixed Model Repeated Measure) is carried out exploratory.”

12b) CONSORT: Methods for additional analyses, such as subgroup analyses and adjusted analyses

No additional analyses in our study.

RESULTS

13a) CONSORT: For each group, the numbers of participants who were randomly assigned, received intended treatment, and were analysed for the primary outcome

Patients’ Demographic and Clinical Characteristics
"A total of 93.3% of patients (14/15) completed all 16 sessions of the vCBT program and participated in the intervention throughout its duration; two patients missed one session each due to adverse events (see below). No patients in the vCBT group were excluded from the analysis. One patient in the TAU group did not report receiving regular medical treatment at week eight and was excluded from the analysis. Finally, 15 patients in the vCBT and 14 patients in the TAU group were analyzed. "

13b) CONSORT: For each group, losses and exclusions after randomisation, together with reasons

"The recruitment process resulted in a total of 38 applications for participation. Three were excluded due to dementia (n = 1), autism spectrum disorder (n = 1), suspected intellectual disability (n = 1), and five declined to participate. As a result of the eligibility assessment, 30 patients were eventually enrolled and randomized."

13b-i) Attrition diagram

In our study, this item is not applicable because face-to-face intervention using video-conference system.

14a) CONSORT: Dates defining the periods of recruitment and follow-up

"We recruited participants through web-based and newspaper advertisements from April 2018 to November 2019." it wrote in methods section. There were not follow-up.

14a-i) Indicate if critical “secular events” fell into the study period

14b) CONSORT: Why the trial ended or was stopped (early)

it is not applicable in our study.

15) CONSORT: A table showing baseline demographic and clinical characteristics for each group

"After enrollment, we assessed the baseline characteristics of the patients sex, age, education, marital status, comorbidity, employment status, age at onset of pain, duration of pain, and treatment history before they entered the intervention period (shown in Table 2). "

15-i) Report demographics associated with digital divide issues

In our study, this item was not applicable.

**16a) CONSORT: For each group, number of participants (denominator) included in each analysis and whether the analysis was by original assigned groups**		
**16-i) Report multiple "denominators" and provide definitions**		
"A total of 93.3% of patients (14/15) completed all 16 sessions of the vCBT program and participated in the intervention throughout its duration; two patients missed one session each due to adverse events (see below). No patients in the vCBT group were excluded from the analysis. One patient in the TAU group did not report receiving regular medical treatment at week eight and was excluded from the analysis. Finally, 15 patients in the vCBT and 14 patients in the TAU group were analyzed. "		
**16-ii) Primary analysis should be intent-to-treat**		
**17a) CONSORT: For each primary and secondary outcome, results for each group, and the estimated effect size and its precision (such as 95% confidence interval)**		
”Primary Outcomes<br>Table 3 shows the adjusted mean reductions of NRS in vCBT and TAU at 16 weeks (primary outcome) and at 8 weeks (secondary outcome) from the baseline. No significant difference was found between the two groups in terms of changes in composite NRS scores at week 16 from baseline (p = 0.357). Table 4 shows raw data on means and standard deviations of NRS scores in vCBT and TAU at 16 weeks and 8 weeks. ”<br><br>”Secondary Outcomes<br>Tables 5 and 6 show the results of efficacy on secondary outcomes. No significant difference was found between the two groups regarding changes in maximum, minimum, and usual NRS scores at week 16 from the baseline. In addition, there was no significant difference in the changes in all NRS scores at week 8 from baseline.<br>Comprehensive pain score: The adjusted mean reduction of the total BPI score in vCBT was significantly larger than that of TAU at 16 weeks (-1.43 [95% CI -2.49 to -0.37], df = 24, p = 0.010). There was no significant difference between the two groups in the adjusted mean reduction of BPI pain severity at 16 weeks. The adjusted mean reduction of BPI pain interference in vCBT was significantly larger than that of TAU at 16 weeks (-1.95 [95% CI -3.33 to -0.56], df = 24, p = 0.008). There was no significant difference between the two groups in the adjusted mean reduction of BPI total score, pain severity, and pain interference at eight weeks.<br>Disability: The adjusted mean reduction of the PDAS score in vCBT was significantly larger than that of TAU at 16 weeks (-9.42 [95% CI -14.47 to -4.36], df = 25, p = 0.001). There was no significant difference between the two groups in the adjusted mean reduction of PDAS at eight weeks.<br>Catastrophizing cognition: The adjusted mean reduction of the PCS score in vCBT was larger than that of TAU at 16 weeks, although the difference was not statistically significant (-6.25 [95% CI -12.89 to 0.38], df=25, p = 0.065). There was no significant difference between the two groups in the adjusted mean reduction of PCS at eight weeks.<br>Depression and anxiety: There was no significant difference between the two groups in the adjusted mean reduction of BDI-II, PHQ-9, and GAD-7 at 16 weeks and 8 weeks, respectively.<br>Health-related quality of life: The adjusted mean change of the EQ-5D-5L index score in vCBT was larger than that of TAU at 16 weeks, although the difference was not statistically significant (0.09 [95% CI -12.89 to 0.38], p = 0.06). There was also no significant difference between the two groups in the adjusted mean change of the EQ-5D-5L index score at eight weeks.”		
**17a-i) Presentation of process outcomes such as metrics of use and intensity of use**		
**17b) CONSORT: For binary outcomes, presentation of both absolute and relative effect sizes is recommended**		
In our study, we did not use binary as outcome.		
**18) CONSORT: Results of any other analyses performed, including subgroup analyses and adjusted analyses, distinguishing pre-specified from exploratory**		
In our study, any other analyses including subgroup analyses and adjusted analyses did not carried out.		
**18-i) Subgroup analysis of comparing only users**		
**19) CONSORT: All important harms or unintended effects in each group**		
"Adverse Events<br>In this study, four adverse events were reported by four different patients in the vCBT group. The first patient was hospitalized due to worsening Bechet's disease, and declined to participate in the study for the fourth session. The second patient had sudden difficulty in opening his eyes due to medically unexplained eyelid pain, and declined to participate in the study for the fifth session. The third patient had a common cold. The fourth patient had temporomandibular joint disorders.<br>Table 2 shows the demographic data of the patients. There were no significant differences between vCBT and TAU in age, gender, length of education, employment status, and number of families living together (p=0.236, p =1.000, p =0.725, p =0.700, p =0.734). Duration of illness in vCBT (M = 11.03 years, SD = 12.64) was significantly longer than that in TAU (M = 7.56 years, SD = 5.84). In both groups, more than 60% of the patients were women. Most of the patients in both groups had an education period of 12 years or more (high school graduate or higher) and were not significantly different (vCBT = 73%, TAU = 57%). At baseline, almost 30% of the patients did not work, be it full-time or part-time (vCBT = 26%, TAU = 35%). Patients were living with at least one family, and only one vCBT patient lived alone. The most reported site of chronic pain was lower back pain. Many patients had orthopedic pain, while others had oral pain such as tongue pain, toothache, and general pain such as rheumatism and fibromyalgia."		
**19-i) Include privacy breaches, technical problems**		
**19-ii) Include qualitative feedback from participants or observations from staff/researchers**		
DISCUSSION		
**20) CONSORT: Trial limitations, addressing sources of potential bias, imprecision, multiplicity of analyses**		
**20-i) Typical limitations in ehealth trials**		
"This study has several limitations. First, the sample size was relatively small. In addition, this study was performed as a single-center study at our hospital. In the near future, large-scale multicenter trials are necessary. Second, further studies targeting patients with specific types of chronic pain will be required to examine the effectiveness of this vCBT program. Third, because we did not use a psychological placebo group as a control condition, we were unable to control non-specific factors and unravel the concrete effects of the vCBT program. Finally, the lack of follow-up data limited the generalization of the conclusions. Long-term follow-up studies should be conducted in the future."		
**21) CONSORT: Generalisability (external validity, applicability) of the trial findings**		
**21-i) Generalizability to other populations**		
**21-ii) Discuss if there were elements in the RCT that would be different in a routine application setting**		
**22) CONSORT: Interpretation consistent with results, balancing benefits and harms, and considering other relevant evidence**		
**22-i) Restate study questions and summarize the answers suggested by the data, starting with primary outcomes and process outcomes (use)**		
To our knowledge, this study is the first RCT to evaluate the effectiveness of vCBT for chronic pain. In addition, we performed a cost-effectiveness analysis of the CBT program using ICER. "Although composite pain intensity by the NRS did not change, vCBT significantly improved the total BPI score, especially pain interference and disability in daily life. Furthermore, in the medical economic evaluation, although our results showed no statistically significant difference, it has been suggested that vCBT may be more cost-effective than TAU for various reasons we discuss in the following sections.<br>The latest systematic review from 75 RCTs about the effectiveness of face-to-face CBT has shown that CBT versus TAU at the treatment end for pain, disability, and distress, showed small effect size, and versus active treatment showed few effects (n = 9401) [9]. "		
**22-ii) Highlight unanswered new questions, suggest future research**		
"Further research is needed to examine augmentation strategies, including an examination of the components of CBT that are best suited for different types of pain. Furthermore, while the present study indicated the cost-effectiveness of this treatment within a small sample, this needs to be verified with a larger sample size."		
Other information		
**23) CONSORT: Registration number and name of trial registry**		
"Trial Registration: University Hospital Medical Information Network: UMIN000031124."		
**24) CONSORT: Where the full trial protocol can be accessed, if available**		
In this study, it is not applicable because our intervention is fece-to-fece CBT.		
**25) CONSORT: Sources of funding and other support (such as supply of drugs), role of funders**		
"Funding for this study was provided by the Health and Labor Sciences Research Grant (H29, Refractory, General, 062). The funding body had no role in the design of the study, collection, analysis, and interpretation of data and manuscript writing."		
**X26-i) Comment on ethics committee approval**		
Ethics and Dissemination<br>"This study was conducted with the approval of the Institutional Review Board of Chiba University Hospital (approval ID number: G29049). In addition, the Clinical Research Ethics Review Committee oversaw the proper implementation of the test at least once a year. The trial registration number was University Hospital Medical Information Network: UMIN000031124."<br>The patients willing to participate in this study were informed of the study objectives and were asked for their consent to participate. Each patient was informed that participation was voluntary and full anonymity would be provided. Each patient was required to provide written consent for participation.		
**x26-ii) Outline informed consent procedures**		
"Written informed consent was obtained face-to-face from all patients after they were fully briefed on the procedure."		

**X26-iii) Safety and security procedures**		
"System Safety<br>In this study, we adopted the ISO 27001-certified Cisco WebEx as the internet conference system. Countermeasures against unauthorized access, information leakage, etc., were taken, and safety problems were cleared. "		
**X27-i) State the relation of the study team towards the system being evaluated**		
